# Laryngeal amyloidosis: a single-centre four-patient case series highlighting diagnostic challenges and clinical heterogeneity

**DOI:** 10.1093/jscr/rjag854

**Published:** 2026-09-28

**Authors:** Ruben Raj Ellan Govan, Aparajith Sathish Kumar, Abdur Rahmaan, Ahmed Youssef, Jaiganesh Jai Manickavasagam

**Affiliations:** Department of Otolaryngology, Ninewells Hospital, NHS Tayside, Ninewells Avenue, Dundee DD2 1GZ, United Kingdom; School of Medicine, University of Dundee, Ninewells Hospital, Ninewells Avenue, Dundee DD2 1GZ, United Kingdom; School of Medicine, University of Dundee, Ninewells Hospital, Ninewells Avenue, Dundee DD2 1GZ, United Kingdom; Department of Otolaryngology, Ninewells Hospital, NHS Tayside, Ninewells Avenue, Dundee DD2 1GZ, United Kingdom; Department of Otolaryngology, Ninewells Hospital, NHS Tayside, Ninewells Avenue, Dundee DD2 1GZ, United Kingdom

**Keywords:** laryngeal amyloidosis, dysphonia, subglottic lesion, amyloidosis, otolaryngology, case series

## Abstract

Laryngeal amyloidosis is a rare condition that may mimic benign, malignant or functional laryngeal disorders. We present a single-centre four-patient case series demonstrating clinical heterogeneity in anatomical distribution, amyloid typing, systemic association and management. All four cases were confirmed histologically. Two patients had localized AL laryngeal amyloidosis following specialist assessment, one patient had laryngotracheal amyloid deposition in the context of known multiple myeloma, and one patient had untyped laryngeal amyloidosis involving the bilateral ventricles and vocal cords. Management varied from conservative airway surveillance to endoscopic debridement, guided by symptom burden, anatomical extent, airway risk and systemic context. This series highlights that the value of reporting multiple cases lies not only in rarity, but in comparing consistent diagnostic principles with divergent disease behaviour and treatment pathways.

## Introduction

Amyloidosis is a heterogeneous group of disorders characterized by deposition of insoluble amyloid fibrils in tissues. Laryngeal involvement is rare, representing <2% of benign laryngeal lesions [1]. Most cases are localized, but systemic amyloidosis may occasionally manifest in the larynx. Clinical presentation commonly includes hoarseness, but lesions can mimic malignancy, inflammatory disease, benign nodules or functional voice disorders [2, 3]. Diagnosis is established histologically, typically with Congo red staining and polarized light birefringence.

The value of this case series lies in comparing four patients with laryngeal amyloidosis who differed in anatomical site, amyloid typing, systemic association and management. We therefore present the cases alongside an expanded comparative table focusing on histological confirmation, amyloid typing, systemic work-up, final classification and management rationale (Table 1).

**Table 1:** Expanded comparison of four patients with laryngeal amyloidosis.

Characteristic	Case 1	Case 2	Case 3	Case 4
Age/Sex	41 F	48 F	84 M	81 F
Presentation	Progressive hoarseness for 6 months; later fatigue and mild exertional dyspnoea	Hoarseness refractory to proton pump inhibitor therapy; later exertional dyspnoea	Incidental subglottic/tracheal lesion during intubation; later mild dyspnoea and dental-related dysphagia	Progressive dysphonia progressing to near-complete aphonia
Anatomical site	Anterior commissure, right aryepiglottic fold/vocal cord region	Subglottis extending into upper trachea	Subglottis/upper trachea	Bilateral vocal cords, laryngeal ventricles and anterior commissure
Histological confirmation	Congo red-positive amyloid with apple-green birefringence; no dysplasia or malignancy	Congo red-positive laryngeal amyloidosis; no vasculitis, dysplasia or epithelial malignancy	Congo red-positive amyloid deposition in subglottic/anterior tracheal wall lesion; no malignancy	Strong Congo red positivity with apple-green birefringence in bilateral ventricular/anterior commissure biopsies
Amyloid typing	Specialist amyloidosis centre review confirmed AL kappa amyloid; local kappa/lambda interpretation limited by artefact	Local kappa/lambda ISH showed no light-chain restriction; specialist assessment concluded localized AL amyloidosis	Formal amyloid typing not documented in available records; attributed clinically to plasma cell dyscrasia/myeloma context	Amyloid typing not performed
Systemic work-up	No plasma cell dyscrasia; no cardiac involvement on echocardiography/biomarkers; normal serum free light chain ratio and no paraprotein/Bence Jones protein reported	No systemic involvement on specialist assessment; no monoclonal band; mildly raised serum free kappa and kappa:lambda ratio; no systemic chemotherapy indicated	Known multiple myeloma with markedly abnormal serum free light chains and renal dysfunction; haematology considered amyloid secondary to myeloma	Routine bloods, renal and liver function, albumin, calcium, eGFR, BNP and CT documented; formal amyloid haematology work-up not documented in available records
Final classification	Localized AL kappa laryngeal amyloidosis	Localized AL laryngeal/subglottic amyloidosis with upper tracheal extension	Laryngotracheal amyloid deposition associated with systemic plasma cell dyscrasia/multiple myeloma	Untyped laryngeal amyloidosis involving both ventricles and vocal cords; no recurrence at latest ENT follow-up
Management rationale	Symptomatic local laryngeal disease; ENT/haematology surveillance with microlaryngoscopy/removal according to symptom burden	Subglottic/tracheal disease with symptomatic progression; urgent endoscopic microdebridement performed, no systemic therapy indicated	No significant airway compromise and high frailty/comorbidity burden; conservative airway surveillance	Severe dysphonia but interval improvement after biopsy; conservative management with PPI, voice therapy and surveillance

**Abbreviations:** BNP, B-type natriuretic peptide; CT, computed tomography; eGFR, estimated glomerular filtration rate; ENT, ear, nose and throat; ISH, *in situ* hybridization; PPI, proton pump inhibitor.

## Case presentations

A comparative summary of histology, amyloid typing, systemic work-up, final classification and management rationale is provided in Table 1.

### Case 1

A previously healthy 41-year-old woman, with no history of smoking and minimal alcohol intake, presented with a six-month history of progressive hoarseness. Flexible nasal endoscopy demonstrated swellings arising from the anterior commissure and right aryepiglottic fold (Fig. 1). Contrast-enhanced computed tomography (CT) of the neck identified a faintly enhancing 7 × 9 mm nodule at the anterior commissure, asymmetry of the right epiglottic fold and adenoidal hypertrophy, without pathological cervical lymphadenopathy.

**Figure 1:**
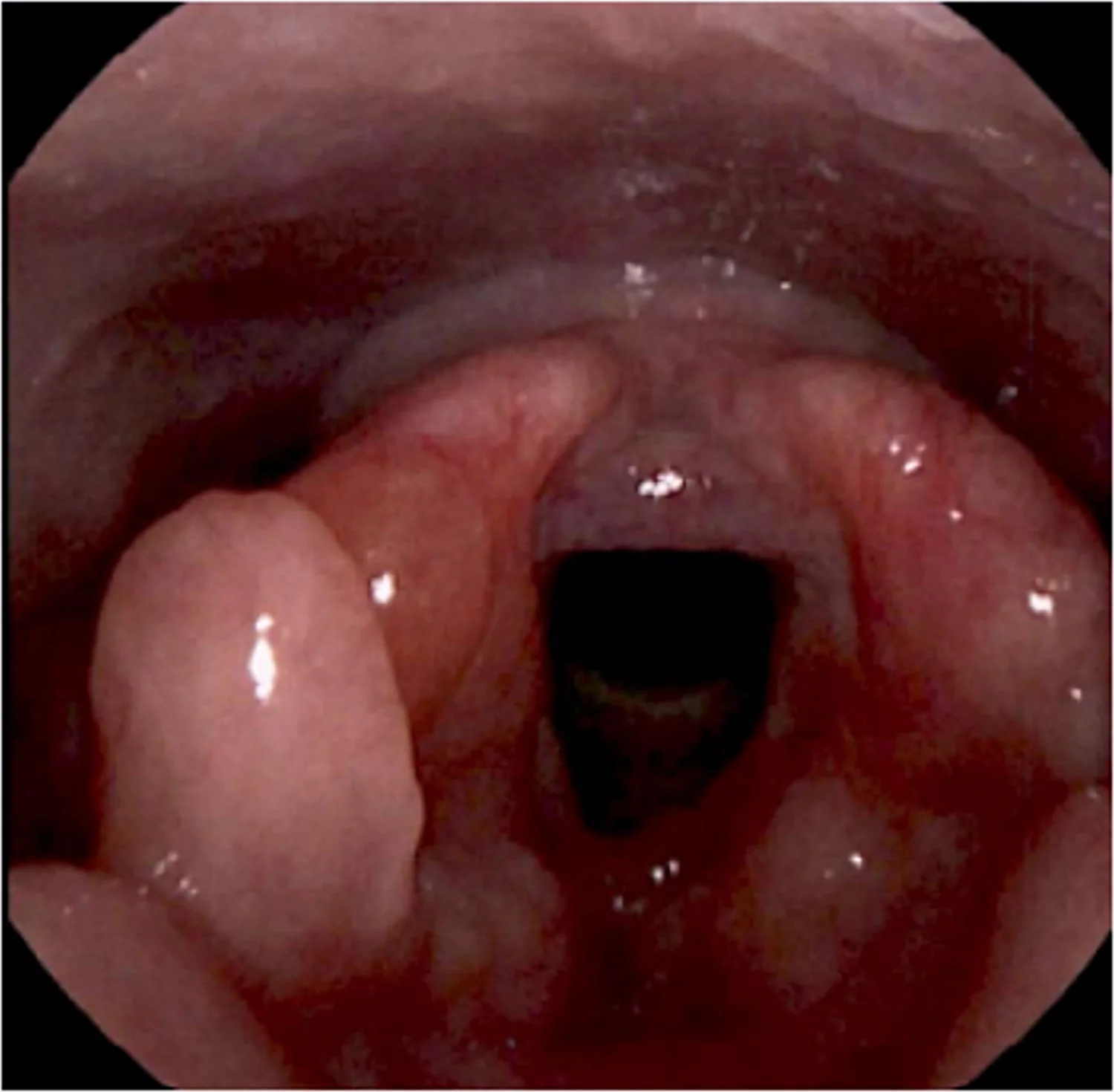
Flexible nasendoscopic image from Case 1 demonstrating a laryngeal lesion involving the anterior commissure/right supraglottic region, subsequently confirmed on biopsy to represent Congo red-positive amyloid deposition.

Histopathological examination of the right aryepiglottic lesion showed amorphous eosinophilic stromal deposits with Congo red positivity and apple-green birefringence under polarized light. There was no dysplasia or malignancy. Local kappa and lambda assessment was limited by artefactual staining. Subsequent specialist amyloidosis centre review confirmed AL kappa amyloid, with no evidence of plasma cell dyscrasia and no cardiac involvement on echocardiography or biomarkers. She was therefore classified as having localized AL kappa laryngeal amyloidosis. Management consisted of ENT and haematology surveillance, with microlaryngoscopy and further removal considered according to symptom burden and lesion persistence.

### Case 2

A 48-year-old woman presented with a seven-month history of hoarseness, with minimal improvement despite high-dose proton pump inhibitor therapy for presumed reflux laryngitis. She was a lifelong non-smoker with no significant comorbidities. She subsequently developed exertional breathlessness, particularly when climbing stairs. Videolaryngoscopy revealed left vocal cord paresis and a subglottic lesion (Fig. 2). Examination under anaesthesia demonstrated a C-shaped soft subglottic mass extending approximately one inch into the trachea, without airway compromise at the time of surgery.

**Figure 2:**
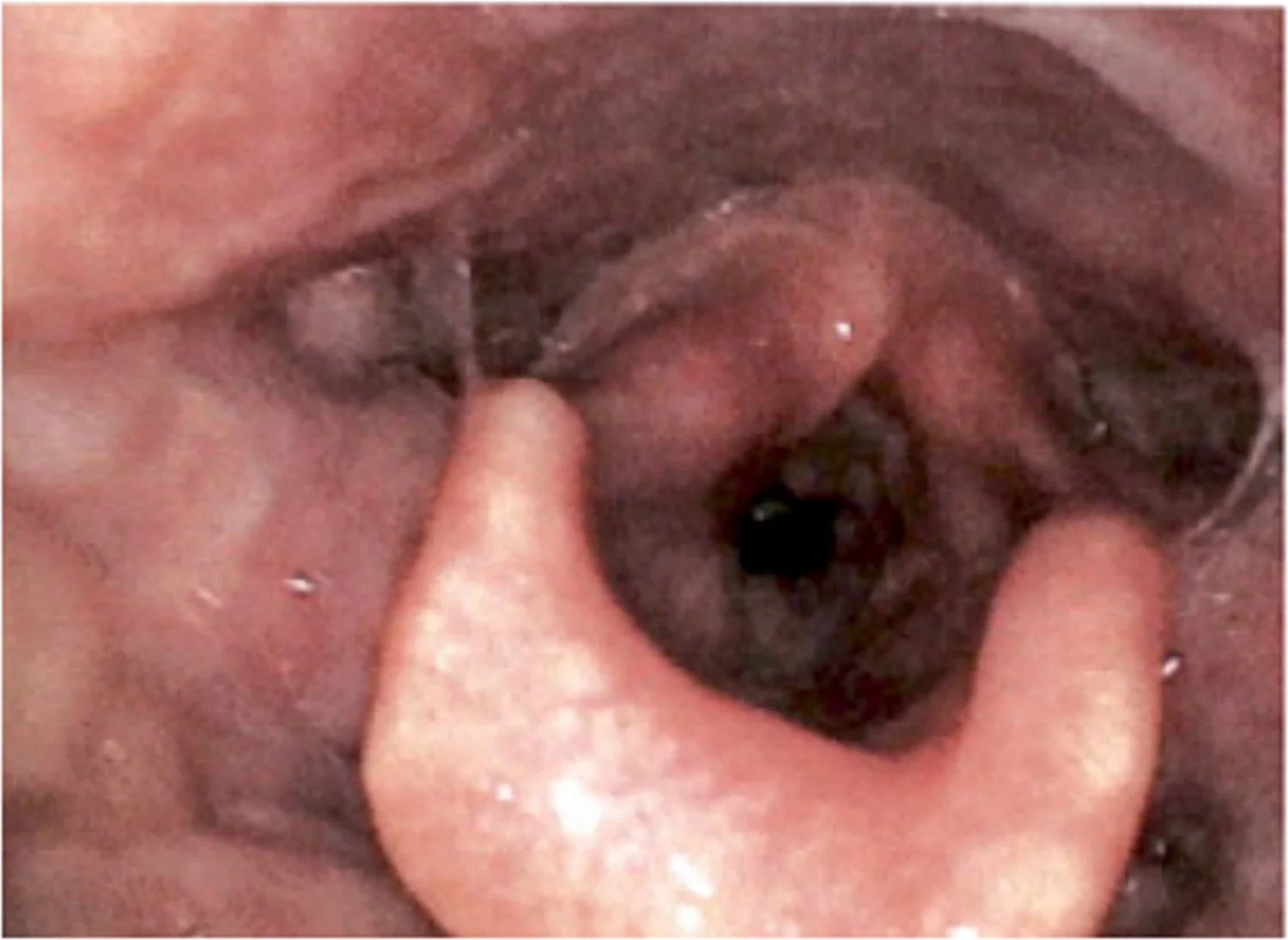
Endoscopic appearance from Case 2 showing a subglottic lesion extending towards the upper trachea. Biopsy confirmed amyloid deposition, with no airway compromise at the time of microlaryngoscopy.

Biopsy showed acutely inflamed respiratory and squamous mucosa with subepithelial amorphous eosinophilic material that stained positively with Congo red, consistent with laryngeal amyloidosis. There was no vasculitis, dysplasia or epithelial malignancy, and local kappa/lambda ISH did not demonstrate light-chain restriction. Systemic assessment showed no monoclonal band, although serum free light chain testing demonstrated a mildly raised kappa level and kappa:lambda ratio. Specialist haematology/amyloidosis review concluded that this represented localized AL amyloidosis with no systemic involvement. With symptomatic progression, she underwent urgent endoscopic microdebridement of laryngeal amyloid deposits in June 2026 without complication. No systemic chemotherapy was indicated.

### Case 3

An 84-year-old man with known multiple myeloma, diabetes mellitus, left ventricular failure and medication-related osteonecrosis of the jaw was incidentally noted to have a polypoid lesion within the larynx during intubation for maxillofacial surgery. He had not reported hoarseness at the time of discovery, although he later described mild exertional dyspnoea and dysphagia, the latter attributed predominantly to dental loss.

Biopsy from the subglottic/anterior tracheal wall lesion showed respiratory-lined mucosa with subepithelial amorphous eosinophilic material positive on Congo red staining, in keeping with amyloid deposition. No malignancy was identified. Formal amyloid typing was not documented in the available records; however, haematology considered the lesion very likely secondary to the patient’s known myeloma. He also had markedly abnormal serum free light chains and renal impairment during follow-up. Given age, frailty, comorbidity and absence of significant airway compromise, management was conservative with airway surveillance. He later died from hospital-acquired pneumonia in the context of IgG light-chain myeloma.

### Case 4

An 81-year-old woman first presented to the ENT service with persistent voice disturbance and an initial impression of possible functional voice disorder. She was later reviewed in the specialist voice clinic with near-complete aphonia. Objective voice assessment using the GRBAS scale demonstrated severe dysphonia (G3), mild roughness (R1), no breathiness (B0), no asthenia (A0) and severe strain (S3). Flexible endoscopic examination with videokymography revealed bulky bilateral lesions involving the vocal cords and laryngeal ventricles, causing significant glottic narrowing (Fig. 3).

**Figure 3:**
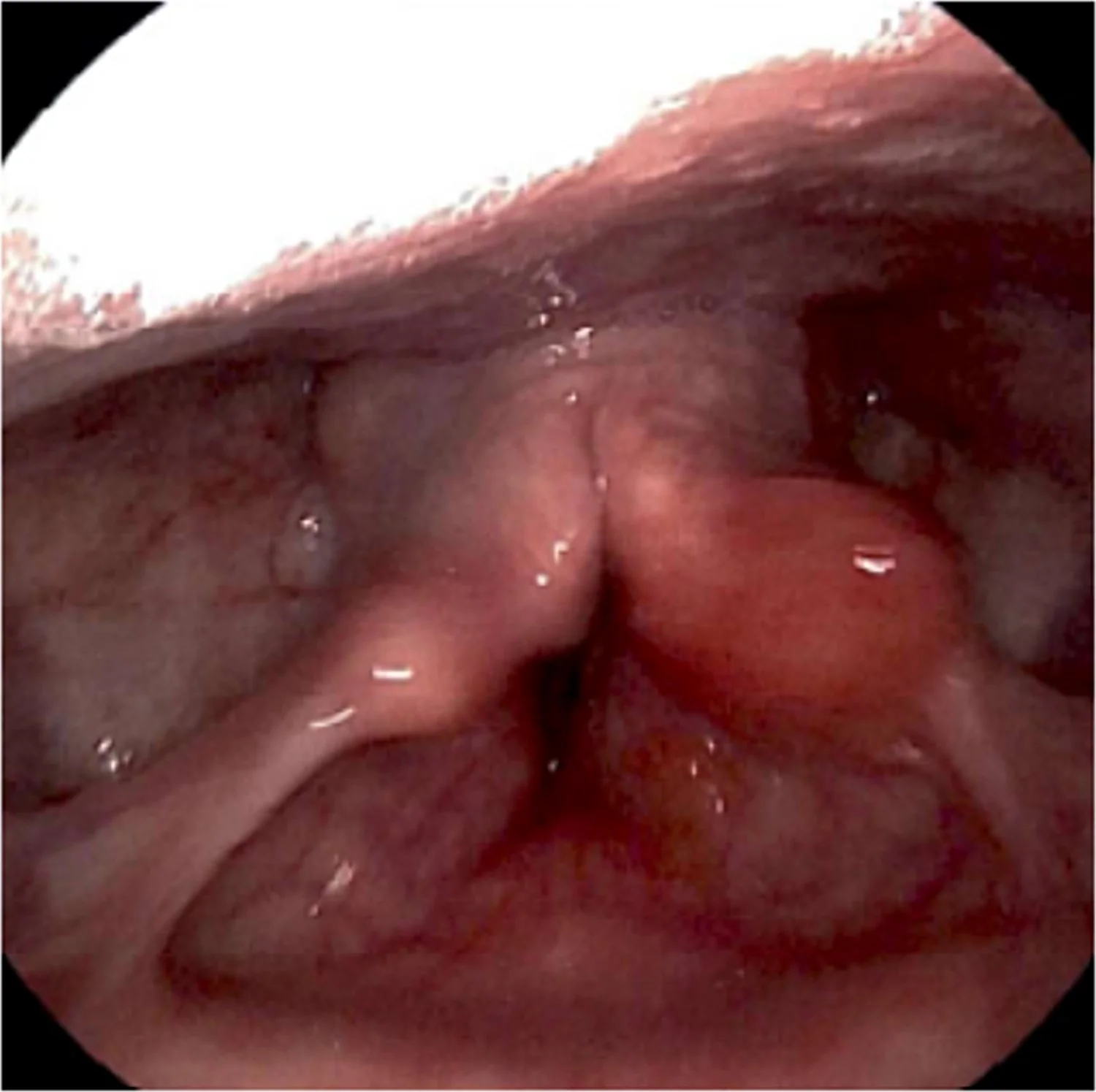
Flexible nasendoscopic image from Case 4 demonstrating bulky bilateral laryngeal ventricular and vocal cord lesions contributing to glottic narrowing. Histopathological analysis confirmed laryngeal amyloidosis.

Urgent microlaryngoscopy demonstrated friable tissue involving both ventricles and the anterior commissure, with differential diagnoses including malignancy and papilloma. Biopsies from the right ventricle, left ventricle and anterior commissure showed dense eosinophilic amorphous deposits, strongly Congo red-positive with apple-green birefringence, confirming laryngeal amyloidosis. Amyloid typing was not performed. Available systemic assessment included routine bloods, renal and liver function, albumin, calcium, estimated glomerular filtration rate, BNP, and CT imaging; however, formal haematological amyloid work-up was not documented in the available records. At latest follow-up, flexible nasendoscopy showed well-healed ventricles and vocal cords, adequate phonation and no recurrence (Fig. 4). She was managed conservatively with proton pump inhibitor therapy, voice strengthening exercises and ongoing surveillance.

**Figure 4:**
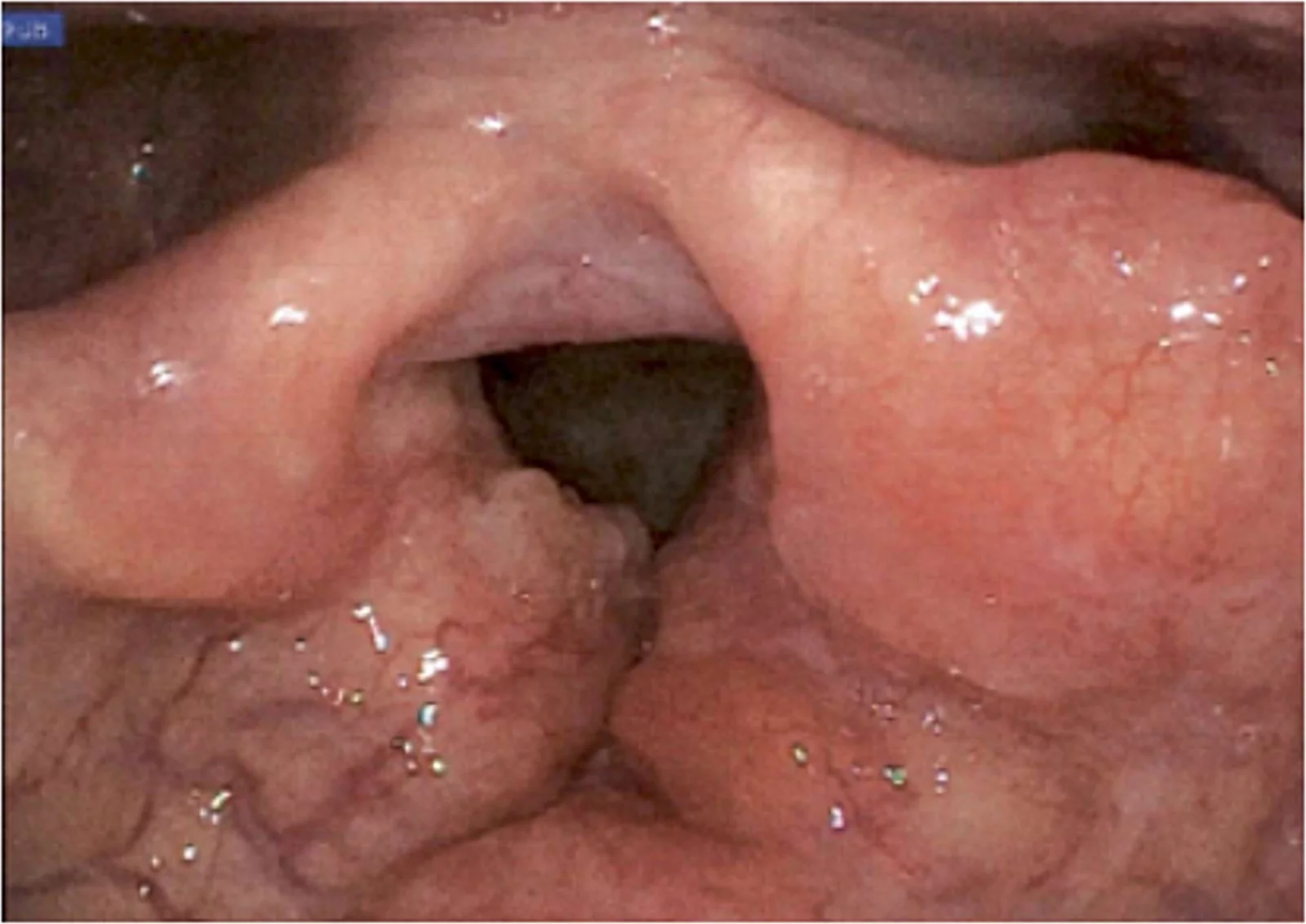
Follow-up endoscopic image from Case 4 demonstrating interval improvement following biopsy and conservative management, with residual fullness but no evidence of acute airway compromise.

## Discussion

### Comparative value of the case series

The strength of this series is not simply that laryngeal amyloidosis is rare, but that four histologically confirmed cases demonstrated distinct clinical patterns. All patients shared tissue confirmation of amyloid deposition in the laryngotracheal complex, yet they differed in anatomical distribution, typing status, systemic association and management pathway. This comparison is clinically important because laryngeal amyloidosis can behave as an isolated local disease, as a locally progressive airway lesion, or as part of systemic plasma cell dyscrasia.

The cases also demonstrate different diagnostic routes. Cases 1 and 2 presented with progressive hoarseness, Case 3 was detected incidentally during intubation, and Case 4 was initially suspected to represent a functional voice disorder before structural disease was identified. These differences reinforce the need to maintain a broad differential diagnosis when evaluating persistent dysphonia or atypical laryngeal lesions. Persistent dysphonia is a common presenting feature of localized laryngeal amyloidosis, although dysphagia and airway symptoms may also occur depending on the site and extent of disease. Laryngeal amyloid may also mimic neoplasia, reinforcing the importance of histological confirmation [4–7].

### Histological confirmation and amyloid typing

Histology was decisive in all four cases. Congo red positivity and apple-green birefringence were documented in Cases 1 and 4, while Cases 2 and 3 also demonstrated Congo red-positive amyloid deposition. Importantly, the cases differed in the completeness of amyloid typing. Case 1 underwent specialist review confirming AL kappa amyloid. Case 2 had local kappa/lambda ISH without light-chain restriction, but specialist assessment concluded localized AL amyloidosis. Case 3 was not formally typed in the available records but occurred in the setting of established multiple myeloma and was considered secondary to systemic plasma cell dyscrasia. Case 4 was histologically confirmed but untyped.

This distinction is important. Labelling every patient as localized AL amyloidosis without confirming typing would overstate the evidence. The revised classification therefore separates proven AL kappa disease, specialist-classified localized AL disease, myeloma-associated disease and untyped laryngeal amyloidosis.

### Systemic assessment and final classification

Systemic evaluation determined final classification and management. Cases 1 and 2 had specialist assessment excluding systemic involvement, supporting classification as localized AL laryngeal amyloidosis. Case 3 differed fundamentally because of known multiple myeloma, markedly abnormal serum free light chains and haematology opinion that the amyloid deposition was secondary to plasma cell dyscrasia. Case 4 had available routine biochemical, renal, hepatic, calcium and BNP assessment, but formal amyloid typing and full haematological work-up were not documented in the records available. It is therefore classified as untyped laryngeal amyloidosis involving both ventricles and vocal cords, rather than definitively localized AL disease. This distinction between localized and systemic light-chain amyloidosis is clinically important, as localized disease represents organ-confined amyloid deposition and generally carries a low risk of progression to systemic disease [8].

### Anatomical heterogeneity and management rationale

Management varied because anatomical site, symptom burden, airway risk and systemic context differed. Case 1 involved the anterior commissure/aryepiglottic region and was managed with surveillance and possible further endoscopic removal for symptomatic voice impairment. Case 2 involved the subglottis and upper trachea; although initially stable, symptom progression led to microdebridement. Case 3 had subglottic/tracheal amyloid in the context of frailty and systemic myeloma, with no significant airway compromise, so conservative surveillance was appropriate. Case 4 had bilateral ventricular and vocal cord disease causing severe dysphonia, but improved after biopsy and remained recurrence-free at latest follow-up; conservative management with voice therapy and surveillance was therefore reasonable.

Endoscopic excision or debulking using cold steel or laser remains the usual approach for symptomatic disease, while observation may be appropriate where symptoms are minimal or airway compromise is absent [9]. Long-term follow-up remains necessary, as recurrence has been reported after primary surgery [10].

### Role of multidisciplinary care

This series highlights the central role of multidisciplinary care in the diagnosis and management of laryngeal amyloidosis. ENT surgeons, pathologists, haematologists, speech and language therapists, maxillofacial surgeons and specialist amyloidosis services all contributed to diagnosis, classification and management. Such collaboration is essential to confirm amyloid deposition, determine typing where possible, exclude systemic disease, guide treatment decisions and optimize long-term outcomes.

## Conclusion

This four-patient case series demonstrates that laryngeal amyloidosis is best understood through comparison of histological confirmation, amyloid typing, systemic assessment, anatomical distribution and management rationale. Two patients were classified as localized AL laryngeal amyloidosis, one had laryngotracheal amyloid associated with systemic plasma cell dyscrasia, and one had untyped laryngeal amyloidosis involving both ventricles and vocal cords. The cases emphasize that persistent dysphonia and atypical laryngeal lesions require biopsy, careful systemic assessment and long-term multidisciplinary follow-up.

## Data Availability

The data underlying this article cannot be shared publicly due to patient confidentiality. Relevant anonymised clinical details are included within the article.

## References

[1] Mesolella M, Petruzzi G, Buono S et al. Focus on localized laryngeal amyloidosis: management of five cases. Open Med (Wars) 2020;15:327–32. 10.1515/med-2020-0400 PMC7712266 33335993

[2] Pai KK, Omiunu AO, Llerena PA et al. Localized laryngeal amyloidosis: a systematic review. Am J Otolaryngol 2022;43:103550. 10.1016/j.amjoto.2022.103550 35917657

[3] Satpathy A, Dasgupta A, Dutta C et al. Isolated laryngotracheal amyloidosis with rare clinical presentation and latest surgical management: case report and review of literature. Int J Head Neck Surg 2024;14:68–70. 10.5005/jp-journals-10001-1558

[4] Knoll RM, Sadow PM, Naunheim MR. Laryngeal amyloidosis. Mayo Clin Proc 2024;99:697–8. 10.1016/j.mayocp.2023.11.018 PMC12204205 38551542

[5] Chow V, Gardner K, Howlett D. Primary localized laryngeal amyloidosis presenting with dysphonia: a case report. J Surg Case Rep 2012;2012:rjs005. 10.1093/jscr/rjs005 PMC3852952 24968392

[6] Bouchlarhem S, Sbai AA, Benfadil D et al. Laryngeal amyloidosis mimicking neoplasia: a case treated by transoral CO2 laser excision. Otolaryngol Case Rep 2025;37:100695. 10.1016/j.xocr.2025.100695

[7] Yiotakis I, Georgolios A, Charalabopoulos A et al. Primary localized laryngeal amyloidosis presenting with hoarseness and dysphagia: a case report. J Med Case Rep 2009;3:9049. 10.4076/1752-1947-3-9049 PMC2767149 19918287

[8] Martínez JC, Lichtman EI. Localized light chain amyloidosis: a self-limited plasmacytic B-cell lymphoproliferative disorder. Front Oncol 2022;12:1002253. 10.3389/fonc.2022.1002253 PMC9705961 36457485

[9] Harris G, Lachmann H, Hawkins P et al. One hundred cases of localized laryngeal amyloidosis - evidence for future management. Laryngoscope 2021;131:E1912–7. 10.1002/lary.29320 33434319

[10] Galluzzi F, Garavello W. Surgical treatment of laryngeal amyloidosis: a systematic review. Eur Arch Otorhinolaryngol 2023;280:3065–74. 10.1007/s00405-023-07881-6 36790723

